# Exploring the emerging technologies and trends of infectious diseases in the post-epidemic era

**DOI:** 10.3389/fpubh.2025.1584938

**Published:** 2025-06-11

**Authors:** Fan Huaiyan, Qian Yuan, He Zhijian, Liu Long, Wang Mei

**Affiliations:** Zhaotong First People's Hospital, Zhaotong, China

**Keywords:** infectious diseases department, post-pandemic era, bioinformatics, artificial intelligence, big data, digital transformation

## Abstract

This study focuses on the development of the infectious diseases department in the post-pandemic era. It reviews the impact, transformation needs, and challenges brought by the pandemic to this field. By discussing the application prospects of emerging technologies such as bioinformatics, artificial intelligence, and big data in epidemic analysis, pathogen research, and medical services, this article demonstrates how interdisciplinary technologies can promote the digital transformation of the infectious diseases department. Meanwhile, it analyzes the development trends of technologies in disease prevention, early diagnosis, innovative treatment methods, and vaccine development. Through case studies and empirical analysis, it reveals the effectiveness of data-driven decision-making in optimizing the management of the infectious diseases department. The research findings indicate the development direction of the infectious diseases department in the post-pandemic era and provide theoretical guidance and technical references for related research and practice.

## 1 Introduction

Globally, there is a growing focus on the development of the infectious diseases department. Especially after the pandemic, people pay more attention to the emerging technologies and trends in this field. With the continuous progress and innovation of science and technology, the infectious diseases department has also to improve professionalism. This article explores the emerging technologies and trends of the infectious diseases department in the post-epidemic era, aiming to provide readers with a comprehensive and in-depth understanding.

Facing unprecedented challenges, the infectious diseases department urgently needs the support of emerging technologies. The pandemic has had a huge impact on the global medical system and has also accelerated the innovation and transformation of medical technology. Therefore, it is necessary to deeply understand the new trends in the development of the infectious diseases department in the post-epidemic era and how emerging technologies can be applied in clinical practice (1, 2).

In addition to traditional treatment methods, emerging technologies are playing an increasingly important role in the field of infectious diseases. The application of technologies such as gene sequencing, artificial intelligence, and big data analysis has brought new possibilities for the diagnosis and treatment of infectious diseases. The continuous breakthroughs of these emerging technologies provide strong support for precision medicine in the infectious diseases department (3, 4).

By deeply exploring the emerging technologies and trends in the development of the infectious diseases department in the post-epidemic era, we can better grasp the development direction of this field and provide useful references for future clinical practice and scientific research. Driven by new technologies, the development of the infectious diseases department is bound to face more challenges and opportunities. We need to be fully prepared to meet the challenges of this new era (5–9).

In conclusion, the emerging technologies and trends in the development of the infectious diseases department in the post-epidemic era are the focus that the medical and scientific research fields urgently need to pay attention to. By deeply exploring and understanding these new technologies and trends, we can provide useful guidance and help for the future development of the infectious diseases department. It is hoped that this article can provide readers with new ideas and perspectives and trigger more discussions and reflections on the development of the infectious diseases department.

## 2 Overview of the post-epidemic era

### 2.1 Impact of the pandemic on the infectious diseases department

In the post-epidemic era, the infectious diseases department, as an important branch of the medical field, has received unprecedented attention and challenges. The impact of the pandemic on the infectious diseases department is self-evident. It has not only accelerated the development of medical technology but also put forward higher requirements and challenges. In this context, exploring the emerging technologies and trends in the development of the infectious diseases department in the post-epidemic era has become a hot topic in the medical community (10, 11).

Firstly, the outbreak of the pandemic has posed new challenges to the diagnosis and treatment model of the infectious diseases department. During the pandemic, the allocation and utilization of medical resources have changed dramatically. The traditional outpatient diagnosis and treatment method has been restricted, while new models such as telemedicine and intelligent diagnosis have emerged rapidly. These new technologies not only provide doctors with more diagnostic and treatment means but also offer patients more convenient medical treatment channels, greatly improving the diagnosis and treatment experience (12, 13).

Secondly, the pandemic has also spurred innovation and breakthroughs in the research field of the infectious diseases department. Facing the challenges of new pathogens, scientific researchers have taken rapid actions, accelerating the progress in vaccine research and development, pathogen monitoring, antiviral drug research, etc. Emerging gene-editing technologies, protein engineering technologies, etc. have opened up new fields for the research of the infectious diseases department and are expected to play an important role in future clinical treatments (3, 4).

In addition, the pandemic has promoted the in-depth integration of the infectious diseases department with other medical fields, forming a new pattern of multidisciplinary cooperation. In the process of fighting the pandemic, it has become a consensus that the infectious diseases department should cooperate closely with clinical medicine, public health, information technology, and other fields (2, 14). Many experts and scholars in the medical community have also called for strengthening the communication and cooperation between different fields to jointly respond to possible new challenges in the future.

In conclusion, the development of the infectious diseases department in the post-epidemic era is full of new opportunities and challenges. With the continuous breakthroughs and innovations of medical technology, the infectious diseases department will also embrace a more glorious development prospect. All of this would not be possible without the unremitting efforts of scientific researchers, the selfless dedication of medical staff, and the high-level attention and support of the whole society for the medical and health cause. May the future medical community work together to contribute more to the health and wellbeing of mankind.

### 2.2 Challenges in the post-epidemic era

As the global pandemic situation is gradually brought under control, people begin to pay attention to the challenges that may be faced in the post-epidemic era. The challenges in the post-epidemic era will involve the development of the infectious diseases department and require emerging technologies and trends to address them.

Firstly, one of the challenges in the post-epidemic era is the problem of pathogen mutation and drug resistance. The mutation of pathogens may lead to changes in the transmission ability or pathogenicity of infectious diseases, thus posing new challenges to prevention and control work. At the same time, the abuse of antibiotics has also led to an increase in drug-resistant bacteria, putting forward higher requirements for the treatment in the infectious diseases department. Therefore, it is necessary to strengthen the monitoring and research on pathogen mutation and drug resistance and seek new breakthroughs in treatment (15, 16).

Secondly, another challenge in the post-epidemic era may be the uneven distribution of medical resources. In the pre-epidemic era, the medical resources in some areas were seriously insufficient, resulting in difficulties in pandemic prevention and control work. In the post-epidemic era, the fair distribution of medical resources will become an important issue. Emerging technologies can make up for the shortage of medical resources through methods such as telemedicine and artificial-intelligence-assisted diagnosis, expand the coverage of medical services, and thus promote the fair distribution of medical resources (12, 17).

In addition, challenges in the infectious diseases department in the post-epidemic era may also include the formulation and implementation of infectious disease prevention and control policies. During the pandemic, different prevention and control policies were adopted in some areas, resulting in incoordination in pandemic prevention and control work. In the post-epidemic era, it is necessary to strengthen international cooperation, unify infectious disease prevention and control policies, and establish a more effective pandemic early-warning and response mechanism to deal with possible new infectious disease threats (2, 18).

In summary, the infectious diseases department faces many challenges in the post-epidemic era, but at the same time, it also breeds many new opportunities. With the help of emerging technologies and trends, we are confident that we can overcome these challenges, promote the development of the infectious diseases department, and make greater contributions to global public health security.

### 2.3 Transformation needs of the infectious diseases department

With the outbreak and global spread of the COVID-19 pandemic, the infectious diseases department faces huge transformation needs in the post-epidemic era. This article explores the challenges and development trends faced by the infectious diseases department during the transformation process, as well as the impact of emerging technologies on its development (11).

In the post-epidemic era, the infectious diseases department not only needs to conduct in-depth research and treatment of traditional diseases but also needs to deal with the challenges of emerging infectious diseases. With the acceleration of globalization, the spread speed and scope of infectious diseases are constantly expanding, which puts forward higher requirements for the infectious diseases department. Therefore, during the transformation process, the infectious diseases department needs to pay more attention to global cooperation and information sharing to deal with the challenges of emerging infectious diseases (10, 19).

Indeed, the transformation needs of the infectious diseases department not only come from the challenges of emerging infectious diseases but also from the continuous update of medical technology and medical models. With the progress of science and technology and the improvement of data analysis capabilities, the infectious diseases department can rely on emerging technologies such as big data and artificial intelligence to better achieve disease prediction, personalized treatment, and disease management (3, 18). Therefore, during the transformation process, the infectious diseases department needs to pay more attention to scientific and technological innovation and interdisciplinary cooperation to adapt to the continuous update of medical models and technologies (1).

In addition, the infectious diseases department also faces challenges in talent team construction and medical resource allocation during the transformation process. Traditional infectious diseases department doctors often need to have rich clinical experience and medical knowledge. However, with the change of the medical model and the progress of technology, infectious diseases department doctors also need to have the ability of interdisciplinary cooperation and information-based management. Therefore, during the transformation process, the infectious diseases department needs to pay more attention to talent cultivation and the rational allocation of medical resources to meet the needs of future medical development.

In conclusion, the infectious diseases department faces huge transformation needs in the post-epidemic era and needs to pay more attention to global cooperation, scientific and technological innovation, and talent team construction. Only by continuously adapting to the development of the times and the changes of the medical model can the infectious diseases department better deal with the challenges of emerging infectious diseases and achieve its own sustainable development (20, 21).

## 3 Overview of emerging technologies

### 3.1 Application of bioinformatics in epidemic analysis

Since the outbreak of the pandemic, bioinformatics technology has played an increasingly important role in epidemic analysis. Bioinformatics technology mainly reveals the laws of biological systems by analyzing large-scale biological data (3, 22).

In epidemic analysis, specialized tools are used for genomic analysis. For example, BLAST (Basic Local Alignment Search Tool) is used to compare pathogen sequences quickly, helping to identify similar pathogens and understand their relationships. MEGA (Molecular Evolutionary Genetics Analysis) is employed to construct phylogenetic trees, which are crucial for tracing the evolutionary paths of viruses. GATK (Genome Analysis Toolkit) is utilized to detect genomic variants, such as single-nucleotide polymorphisms that might be related to drug resistance (23).

Key datasets in this field include GISAID (Global Initiative on Sharing All Influenza Data), which as of 2024, hosts over 15 million viral genome sequences. This real-time data source is invaluable for tracking emerging virus variants (22). Another important dataset is NCBI GenBank, a comprehensive genetic data repository.

During the COVID-19 pandemic, researchers used GISAID data in conjunction with PhyloViZ software. By analyzing the data, they were able to map the global spread of the Omicron variant. They identified 32 spike protein mutations in the Omicron variant through this analysis, which were associated with immune escape. This provided crucial information for understanding the variant’s transmissibility and formulating targeted prevention and control strategies (3, 23).

Bioinformatics can also help scientific researchers conduct virus sequence alignment and evolutionary analysis in epidemic analysis. By comparing the gene sequences of different virus strains, we can reveal the origin, transmission routes, and mutation laws of the virus, providing support for epidemic tracing and epidemiological investigations. Through evolutionary analysis, we can also predict the mutation trend of the virus, providing a reference for subsequent epidemic prevention and control.

Furthermore, bioinformatics technology can also help scientific researchers conduct host genome analysis, revealing the host’s resistance mechanism to pathogens. Through the analysis of host genome data, we can discover gene mutations related to disease susceptibility, providing support for individualized prevention and control and drug research and development. At the same time, it can also reveal the interaction network between the host and the pathogen, providing in-depth understanding of the disease pathogenesis and pathophysiological process.

In conclusion, the application of bioinformatics in epidemic analysis has become an important tool in epidemic prevention and control and scientific research. With the continuous development and improvement of technology, it is believed that bioinformatics technology will play an even more important role in the infectious diseases department in the post-epidemic era, providing stronger support for mankind to overcome diseases.

### 3.2 Combined application of artificial intelligence and big data

In the post-epidemic landscape, the synergistic integration of artificial intelligence (AI) and big data has emerged as a transformative paradigm for infectious disease management. Machine learning algorithms, including random forest classifiers and long short-term memory (LSTM) networks, process heterogeneous datasets-such as electronic health records (EHRs), mobility trajectories, and epidemiological surveillance data-to inform evidence-based decision-making. For example, during Wuhan’s 2020 pandemic response, an LSTM-based predictive model analyzed real-time hospital admission data to forecast ICU bed demand with 94% accuracy, enabling proactive allocation of ventilators and critical care staff (18, 24). In New York, linear programming algorithms optimized the distribution of personal protective equipment (PPE), reducing stockout incidents by 45% while minimizing resource waste-an efficiency unattainable through traditional rule-based approaches (3, 16).

Diagnostically, the deep learning model CheXNet, trained on 150,000 chest X-rays, demonstrates an AUC of 0.93 in detecting COVID-19-associated ground-glass opacities, outperforming junior radiologists in early-stage lesion identification (13, 18). These applications underscore how AI-driven analytics, when coupled with big data, enhance diagnostic precision and operational agility, though challenges related to data privacy and algorithmic interpretability remain critical areas for improvement.

## 4 Analysis of technology development trends

### 4.1 Trends in disease prevention and early diagnosis technologies

With the aggravation and complication of disease transmission, the development of disease prevention and early diagnosis technologies has become an important direction of current medical scientific research. In the future, disease prevention and early diagnosis technologies will play an even more important role in the field of the infectious diseases department. This article analyzes and explores the development trends of disease prevention and early diagnosis technologies.

In terms of disease prevention, with the continuous breakthroughs and progress of gene-detection technologies, individualized prevention will become the future development trend. By obtaining and analyzing individual gene information, we can assess disease susceptibility and then develop personalized prevention plans. At the same time, with the wide application of artificial intelligence technology, intelligent prevention systems will become an important means of disease prevention. Through the analysis and mining of big data, intelligent prevention systems can timely detect the trends of disease outbreaks and take corresponding preventive measures in advance, thus effectively curbing the spread of diseases (17, 18).

In the aspect of early diagnosis technologies, early diagnosis technologies based on biomarkers will become the future development focus. Biomarkers are indicators that reflect the physiological state of the organism, the occurrence, and development process of diseases, and are of great significance for the early diagnosis of diseases. With the continuous development of biochemistry, bioengineering, and nanotechnology, more and more biomarkers are being discovered and applied to clinical diagnosis. At the same time, image-diagnosis technologies will also be further developed. Medical imaging technologies such as CT and MRI will play a more important role in early disease diagnosis.

In addition, miniaturized and portable diagnostic devices will also become a future development trend. With the continuous maturity of medical device technology, more and more portable diagnostic devices will be put into use, making early diagnosis more convenient and rapid. These devices can play important roles in clinical diagnosis, home healthcare, and other fields and are of great significance for the early screening and diagnosis of diseases (25–27).

In conclusion, the development of disease prevention and early diagnosis technologies in the field of the infectious diseases department is of great significance. In the future, individualized prevention, intelligent prevention systems, the application of biomarkers, the development of image-diagnosis technologies, and the use of miniaturized and portable diagnostic devices will become the main development trends, providing more effective means and technical support for the early diagnosis and prevention of diseases.

### 4.2 New trends in treatment methods and vaccine research and development

During the pandemic, new trends in treatment methods and vaccine research and development have become the focus of attention. With the continuous progress of science and technology, the emergence of emerging technologies has brought new hope for the development of the infectious diseases department. In the exploration of emerging technologies and trends in the development of the infectious diseases department in the post-epidemic era, new trends in treatment methods and vaccine research and development have received much attention.

Firstly, gene-editing technology shows great potential in the treatment of infectious diseases. Through gene-editing technology, scientists can accurately repair or modify genes in patients’ bodies, thereby achieving the goal of treating infectious diseases (23, 28). For example, using the CRISPR gene-editing technology, scientific researchers have successfully developed treatment methods for certain viruses, providing new options for the shortcomings of traditional treatment methods.

Secondly, the field of vaccine research and development is also constantly exploring new technologies and methods. In addition to traditional vaccine research and development methods, vaccine research and development based on genetic engineering technology has become a hot topic. Using genetic engineering technology, scientists can precisely design virus antigens, thus developing more effective vaccines. In addition, the application of nanotechnology has also brought new breakthroughs to vaccine research and development. Nanoparticle vaccines can improve the stability and immunogenicity of vaccines, injecting new vitality into vaccine research and development (4, 22).

In addition, immunotherapy, as an emerging treatment method, has also received increasing attention. Immunotherapy resists infectious diseases by regulating the patient’s immune system and has the characteristics of strong pertinence and few side effects. Currently, immunotherapy has achieved remarkable results in the treatment of certain infectious diseases, bringing new hope for the treatment in the infectious diseases department.

In the new trends of treatment methods and vaccine research and development, the continuous innovation of science and technology has opened up a new situation for the development of the infectious diseases department. With the continuous emergence of new technologies such as gene-editing technology, genetic engineering technology, and immunotherapy, we have reason to believe that in the post-epidemic era, the infectious diseases department will embrace a brighter development prospect. To sum up, the new trends in treatment methods and vaccine research and development will become an important driving force for the development of the infectious diseases department, bringing new hope for humanity to fight against infectious diseases.

### 4.3 The trend of digital transformation of medical service models

In the exploration of emerging technologies and trends in the development of the infectious diseases department in the post-epidemic era, the trend of digital transformation of medical service models has become a highly concerned topic (12, 13). Digital transformation has become a trend in the medical field, which transforms medical services from the traditional offline model to a more convenient and efficient online model. Driven by digital transformation, medical service models present a series of new characteristics and trends.

Firstly, with the rapid development of the Internet and mobile Internet technologies, medical services have gradually shifted online. Patients can make appointments, consult doctors online, and purchase medicines through mobile apps or websites. The emergence of this online medical service model has greatly improved the convenience and efficiency of medical services (6, 19). Patients no longer need to queue up; they can meet their medical needs at home, which is undoubtedly a blessing for busy modern people.

Secondly, digital transformation has also promoted the application of medical big data. The digital transformation of medical service models enables the collection and analysis of a large amount of medical data (18, 29). These data can not only help doctors better understand patients’ conditions and needs but also provide valuable reference materials for medical scientific research. Through the analysis of big data, early warning and accurate diagnosis of diseases can be achieved, improving the quality and efficiency of medical services.

In addition, under the trend of digital transformation, telemedicine services have gradually become an emerging medical model. Patients can remotely consult experts via video or phone. This model not only provides patients with a wider range of choices but also offers doctors more working methods. Especially during the pandemic, telemedicine services have become an important medical means, providing patients with a safe and convenient way to seek medical advice.

Finally, digital transformation has promoted the development of smart medicine. By introducing technologies such as artificial intelligence and big data analysis, smart medical systems can achieve a series of intelligent medical services, such as medical record management, diagnostic assistance, and surgical robots, providing patients with more accurate and safer medical services (32, 33).

To sum up, the digital transformation of medical service models has become a new trend in the medical field and will continue to develop in the future. Digital transformation not only provides patients with more convenient and efficient medical services but also brings new opportunities and challenges to the development of the medical industry. We look forward to more innovations and progress in medical services brought about by digital transformation.

## 5 Case studies and empirical analyses

### 5.1 Application cases of interdisciplinary technologies in epidemic management

The COVID-19 pandemic highlighted the necessity of interdisciplinary solutions in epidemic management, with technologies like unmanned aerial vehicles (UAVs) and blockchain demonstrating tangible real-world impacts. In Rwanda, Zipline’s drone logistics network revolutionized vaccine delivery by transporting over 2 million COVID-19 doses to remote clinics between 2020 and 2022, reducing delivery times from 48 h to 30 min and improving vaccination coverage by 40% (11, 12). This innovation mitigated geographical barriers, offering a scalable model for resource distribution in underserved regions.

Blockchain technology also emerged as a cornerstone for supply chain transparency. IBM’s Blockchain Platform tracked 1.2 million COVID-19 test kits across 20 countries in 2021, reducing logistics delays by 60% and ensuring end-to-end traceability of cold-chain storage conditions (2, 24). By eliminating manual discrepancies and enhancing accountability, such decentralized systems set a new benchmark for global medical resource management during public health crises.

### 5.2 Practices of emerging technologies in the research of difficult cases

In the post-epidemic era, the development of the infectious diseases department faces new opportunities and challenges. Studying difficult cases has always been one of the important tasks of the infectious diseases department, and emerging technologies are gradually becoming powerful tools for studying difficult cases. This article explores the practices of emerging technologies in the research of difficult cases, hoping to provide some new ideas and directions for the development of the infectious diseases department.

Firstly, gene sequencing technology is playing an increasingly important role in the research of difficult cases. Through the sequencing and analysis of patients’ genomes, we can discover some rare gene variations or mutations that may be related to the occurrence of patients’ diseases. The development of gene sequencing technology provides new ideas and methods for the diagnosis and treatment of difficult cases (3, 23).

Secondly, single-cell sequencing technology provides a brand-new perspective for the research of difficult cases. Traditional gene sequencing technology usually only obtains gene expression information at the population level, while single-cell sequencing technology can reveal the gene expression profile of a single cell, helping us better understand the mechanism of disease occurrence and development. Through single-cell sequencing technology, we can discover some important information hidden in traditional sequencing data, providing more accurate basis for the diagnosis and treatment of difficult cases.

In addition, the application of artificial intelligence technology has gradually become a new hotspot in the research of difficult cases. Artificial intelligence technology can help doctors discover some potential laws or characteristics through the analysis of a large amount of medical data, providing auxiliary decision-making for the diagnosis and treatment of difficult cases. At the same time, artificial intelligence technology can also help doctors screen cases and conduct risk assessments, improving the diagnostic efficiency and accuracy of difficult cases.

Then, the application of evolutionary algorithms and machine learning technologies also brings new opportunities for the research of difficult cases. Through the analysis and modeling of case data, evolutionary algorithms and machine learning technologies can help doctors discover some potential causes or pathological mechanisms, providing more clues and directions for the diagnosis and treatment of difficult cases. At the same time, these technologies can also help doctors conduct risk assessments and predictions of cases, guiding clinical decision-making and the formulation of treatment plans.

Finally, the practices of emerging technologies in the research of difficult cases still face some challenges and limitations, which require us to conduct further in-depth research and exploration. For example, the high cost and complex operation of emerging technologies may limit their promotion and application in clinical practice. At the same time, ethical and privacy issues of emerging technologies also need to be carefully considered and resolved. Therefore, while promoting the practices of emerging technologies in the research of difficult cases, we need to strengthen standardization and management to ensure their safety and effectiveness in clinical practice.

To sum up, the practices of emerging technologies in the research of difficult cases will bring new opportunities and challenges to the development of the infectious diseases department. We need to constantly explore and innovate to better apply emerging technologies to the diagnosis and treatment of difficult cases and provide better protection for patients’ health and quality of life.

### 5.3 Data-driven auxiliary decision-making analysis in the infectious diseases department

This article focuses on data-driven auxiliary decision-making analysis in the infectious diseases department and explores the application and trends of this emerging technology in the development of the infectious diseases department in the post-epidemic era through case studies and empirical analyses.

In the context of highly developed information technology, traditional decision-making analysis in the infectious diseases department is gradually developing toward a data-driven direction. Data-driven auxiliary decision-making analysis in the infectious diseases department will analyze information such as the transmission laws of pathogens and the disease dynamics of patients through big data technology, providing a more scientific and accurate basis for decision-making in the infectious diseases department (3, 18, 29).

Taking an infectious diseases department hospital as an example, through in-depth mining and analysis of the data of the infectious diseases department of this hospital, some valuable laws and trends have been discovered. Firstly, data analysis shows that the transmission of a specific pathogen has certain seasonal patterns, which is of great significance for hospital infection control. Secondly, by analyzing patients’ condition data and medication situations, some new treatment plans and strategies have been discovered, which play a positive role in improving treatment effects and reducing medical costs. In addition, the data also shows the flow trajectories and contact networks of the patient group, providing new ideas and methods for the prevention and control work of the infectious diseases department (30).

Data-driven auxiliary decision-making analysis in the infectious diseases department can not only improve medical quality and efficiency but also help hospitals better respond to sudden infectious disease events. After the outbreak of the pandemic, data analysis can timely discover the transmission laws and mutation situations of pathogens, providing important bases for formulating targeted prevention and control strategies. At the same time, through the analysis of the behavior trajectories and spatial distributions of the patient group, it is possible to better trace and manage close contacts, effectively curbing the spread of the epidemic.

To sum up, data-driven auxiliary decision-making analysis in the infectious diseases department is an emerging technology and trend in the development of the infectious diseases department in the post-epidemic era. Through case studies and empirical analyses, we can see the important role and great potential of this technology. In the future, with the continuous development of information technology and the continuous accumulation of medical data, data-driven auxiliary decision-making analysis in the infectious diseases department will be more deeply and widely applied in the field of the infectious diseases department, making greater contributions to the health cause of mankind.

## 6 Ethical implications of emerging technologies

The adoption of advanced technologies in infectious disease management necessitates a robust ethical framework to balance innovation with societal and regulatory considerations (2, 7, 18).

### 6.1 Data ownership and equity

Disputes over genomic data sovereignty, particularly among indigenous communities in regions like the Amazon Basin, highlight the need for equitable governance models. Frameworks such as the NIH Genomic Data Sharing Policy aim to ensure that communities contributing biological data receive fair benefits from research, yet challenges persist in achieving global consensus on data ownership and benefit-sharing mechanisms (10, 24).

### 6.2 AI Bias and transparency

AI algorithms trained on homogenous datasets may exhibit diagnostic biases, as evidenced by a 2022 study showing 18% higher false-negative rates in COVID-19 screening models for African populations due to limited genetic diversity in training data (18, 31). Tools like SHAP (SHapley Additive exPlanations) have become essential for auditing model decisions, enabling researchers to identify and rectify biased outputs. Transparency in AI decision-making is crucial to fostering trust among healthcare providers and patients alike.

### 6.3 CRISPR ethics and dual use

Gene-editing technologies such as CRISPR raise significant ethical concerns regarding unintended genetic modifications and potential dual-use risks. The WHO Governance Framework for Human Genome Editing (2023) advocates strict oversight of clinical trials involving heritable gene edits, emphasizing the need for public participation and scientific responsibility to prevent misuse and ensure ethical deployment (4, 7).

## 7 Comparative table highlighting novel contributions

The following table contextualizes the manuscript’s key technological advancements within the landscape of prior approaches, enhancing clarity on its scholarly contributions (Table 1).

**Table 1:** Comparative analysis of emerging technologies in infectious disease management.

Technology/theme	Prior approaches	Novel insights/advancements in this study
Bioinformatics	Manual sequence alignment; regional genomic databases (1, 23)	Integration of real-time global datasets (GISAID) and AI-augmented tools (GATK, PhyloViZ) for rapid variant characterization and host-pathogen interaction modeling. Use of these in case studies, like analyzing Omicron variant and multi-drug resistant bacteria outbreaks, providing more accurate and timely information for epidemic control (3, 22).
AI-driven resource allocation	Static bed allocation; rule-based heuristics (18, 24)	Development of dynamic LSTM models achieving >90% accuracy in predicting ICU demand (e.g., in Wuhan), enabling real-time redistribution of critical resources during surges. Application of linear programming algorithms to optimize PPE distribution, reducing stockouts and waste (3, 22).
Nanoparticle vaccines	Traditional protein-based adjuvants (4)	Application of nanotechnology to enhance vaccine stability (e.g., mRNA-LNP formulations reducing storage temperature requirements from −80 to 2–8°C) and immunogenicity, revolutionizing vaccine development speed and effectiveness as seen in COVID-19 mRNA vaccines (4, 22).
Blockchain in healthcare	Centralized, paper-based supply chain records (24)	Decentralized blockchain systems improving transparency in PPE/test kit distribution (e.g., 60% reduction in logistics delays in IBM’s global pilot). Ensuring end-to-end traceability of medical resources, which is crucial for efficient epidemic management (2, 24).
Telemedicine	Limited remote consultations; analog data transfer (12, 13)	AI-driven triage systems (e.g., Aarogya Setu app in India triaging 15 million consultations) and blockchain-secured telehealth platforms ensuring data integrity and access equity. Expanding the reach and effectiveness of medical services, especially during pandemics (3, 12).

## 8 Conclusion

The COVID-19 pandemic has underscored the pivotal role of emerging technologies in transforming infectious disease management. From bioinformatics enabling real-time pathogen genomics to AI optimizing resource allocation, these innovations have demonstrated efficacy in addressing post-pandemic challenges. However, ethical considerations-including data sovereignty, AI bias mitigation, and CRISPR governance-remain imperative to ensure responsible and inclusive technological advancement.

Looking forward, interdisciplinary collaboration and global coordination are essential to harnessing the full potential of these technologies. By integrating novel tools with robust ethical frameworks, the infectious diseases department can build resilience, enhance diagnostic precision, and deliver patient-centered care in an era marked by evolving public health threats.

## Data Availability

The original contributions presented in the study are included in the article/supplementary material, further inquiries can be directed to the corresponding author.
